# Hypertensive Anaphylaxis After Moderna COVID-19 Vaccination: A Case Report

**DOI:** 10.7759/cureus.25586

**Published:** 2022-06-01

**Authors:** Yoshitaka Furuto, Takaya Nohara, Hirohisa Hamada, Yuko Shibuya

**Affiliations:** 1 Department of Hypertension and Nephrology, NTT Medical Centre Tokyo, Tokyo, JPN; 2 Department of Emergency Medicine, NTT Medical Centre Tokyo, Tokyo, JPN

**Keywords:** case report, coronavirus, covid-19 vaccination, hypertension, anaphylaxis

## Abstract

Hypotension after exposure to an allergen is a well-known indicator of an anaphylactic reaction. However, hypertensive anaphylaxis often goes unrecognized. Increased blood pressure can present as an anaphylactic reaction, which is called hypertensive anaphylaxis. A 48-year-old woman complained of a tickle sensation in the throat and dyspnea 30 minutes after being administered the first dose of the Moderna coronavirus disease 2019 (COVID-19) vaccine. The patient had no history of hypertension, anxiety, or panic disorder. Forty-five minutes after the vaccination, stridor was noted, and the patient developed severe hypertension with a blood pressure of 197/153 mmHg. The patient also had tachycardia, cervical angioedema, and nausea, which occurred in a short period of time, indicating type I hypersensitivity reaction, that is, an anaphylactic reaction. The patient was diagnosed with Brighton classification Level 1 anaphylaxis caused by COVID-19 vaccination. For managing the patient, two intramuscular adrenaline injections, famotidine, chlorpheniramine, metoclopramide, and methylprednisolone were administered via intravenous infusion. After the administration of medications, all symptoms resolved, and the blood pressure was reduced. Other differential diagnoses for increased blood pressure after vaccination were excluded; therefore, we concluded that this phenomenon of increased blood pressure was hypertensive anaphylaxis. Not only hypotension but also the acute onset of increased blood pressure after vaccination may occur as a premonitory symptom of anaphylaxis. In hypertensive anaphylaxis, both anaphylaxis and increased blood pressure can be treated with intramuscular adrenaline injection. Clinicians should be aware of the occurrence of hypertensive anaphylaxis.

## Introduction

Anaphylaxis is an acute systemic hypersensitivity reaction that may lead to death in healthy individuals immediately after onset, and delayed adrenaline (AD) administration increases the risk of death. Therefore, prompt diagnosis and treatment are important [1]. In anaphylaxis, symptoms may occur in multiple organ systems at the same time or with some delay. Anaphylaxis presents with mucocutaneous symptoms of pruritus, erythema, and urticaria; respiratory symptoms of stridor and dyspnea; gastrointestinal symptoms of vomiting and abdominal pain; central nervous symptoms of anxiety and altered consciousness; and cardiovascular symptoms of tachycardia, bradycardia, hypotension, and arrhythmia. Therefore, these are used in differentiating anaphylaxis from other mimics. Although hypotension is not a prerequisite for diagnosing anaphylaxis, hypotension after exposure to an allergen is a well-known indicator of an anaphylactic reaction; it is the most common cardiovascular system finding and is considered a diagnostic criterion for anaphylaxis [2]. Conversely, although rare, increased blood pressure can be encountered as a result of anaphylactic reactions; however, it is not included in any guidelines [1]. This phenomenon is called hypertensive anaphylaxis (HA) and it has previously been reported in the literature [3].

With the increase in coronavirus disease 2019 (COVID-19) vaccination, healthcare professionals are required to pay attention to anaphylaxis and take appropriate measures. Anaphylaxis is an important adverse reaction and mostly occurs within 15 minutes after vaccination. Its frequency in Moderna COVID-19 vaccination is reported to be 2.8 per one million vaccinations, which is higher than in regular vaccinations; therefore, anaphylaxis is attracting attention in COVID-19 [4,5]. Herein, we report a case of HA that developed after the Moderna COVID-19 vaccination.

## Case presentation

This case involves a 48-year-old woman. She complained of a tickle in her throat and dyspnea 30 minutes after she was administered her first dose of Moderna COVID-19 vaccine. Written informed consent was obtained from the patient for the publication of this case report. The ethics committee of NTT Medical Center, Tokyo, approved the study. The patient shared their perspective on the treatments received, and patient anonymity was preserved. She, as well as her children, had a history of bronchial asthma (BA) without recent exacerbation, but had no history of hypertension (HTN). The patient did not have anxiety or panic disorder, did not drink alcohol or smoke cigarettes, and had no other history of allergies. The patient’s blood pressure (BP) during her medical examination before vaccination was below 140/90 mmHg. The patient’s initial BP measured 30 minutes after administration of vaccination was 163/101 mmHg and was accompanied by the following symptoms: tickle sensation in the throat; dyspnea; heart rate (HR), 85 beats/min; respiratory rate (RR), 18 breaths/min; and saturation of percutaneous oxygen (SpO2), 98%; all of which were associated with the increased blood pressure and were suggestive of an anaphylactic reaction. Her mental condition was stable. Forty-five minutes after vaccination, stridor was noted, and the patient developed severe hypertension with BP 197/153 mmHg, HR 82 beats/min, RR 20 breaths/min, SpO2 98%; tachycardia, cervical angioedema, and nausea gradually developed. The patient was diagnosed with Brighton classification Level 1 anaphylaxis caused by COVID-19 vaccination [6].

**Figure 1:**
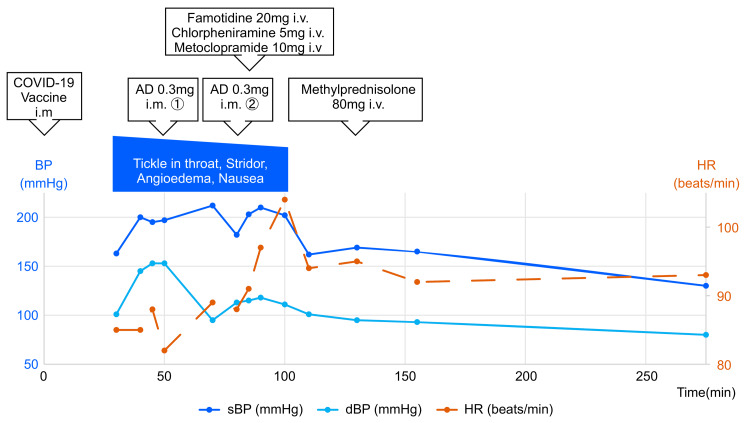
The treatment course, the symptoms, and changes in blood pressure and heart rate of case 1 over time. COVID-19: Coronavirus disease 2019; AD: Adrenaline; BP: Blood pressure; dBP: diastolic blood pressure; HR: Heart rate; i.m: intramuscular injection; i.v: intravenous injection; IV, intravenous; min: minutes; sBP: systolic blood pressure.

Approximately 50 minutes after inoculation, she was administered the first intramuscular AD (0.3 mg) injection and was moved to the emergency room (ER). The physical findings at the ER were as follows (70 minutes after inoculation): body mass index (BMI) 22.5 kg/m^2^; BP 212/95 mmHg; HR 89 beats/min; body temperature (BT) 35.5 °C; RR 20 breaths/min, SpO2 99%; clear and alert. The patient had persistent nausea, conjunctival hyperemia, facial swelling, and nonpruritic angioedema in the neck. She had no significant oral cavity findings, the stridor disappeared, and there were no other special notes. There were no abnormalities in the blood and urine tests, electrocardiogram, and chest X-ray. At approximately 80 minutes after vaccination, the tickle sensation in the throat, angioedema, and nausea persisted; therefore, a second intramuscular AD (0.3 mg) injection was administered. Furthermore, 20 mg famotidine, 5 mg chlorpheniramine, 10 mg metoclopramide, and 80 mg methylprednisolone were administered via intravenous (IV) infusion. Approximately 110 minutes after vaccination, all her symptoms disappeared, her systolic BP reduced to 160 mmHg, and she was hospitalized for observation. Approximately 275 minutes after vaccination, her BP reduced to 130/80 mmHg (the therapeutic progress is shown in Figure 1). The next morning, she was discharged with a BP of 132/84 mmHg and was in good condition without a biphasic reaction.

## Discussion

From June 28 to October 28, 2021, the Moderna COVID-19 vaccine was administered 50,477 times at our hospital during our workplace vaccination program. The World Health Organization (WHO) and regulatory authorities recommend the use of the Brighton Collaboration Anaphylaxis Working Group classification of anaphylaxis for pharmacovigilance registers [6]. Nine patients developed anaphylaxis during the vaccination program based on the Brighton classification. In the present report, we defined HA as the acute onset of increased blood pressure above 140/90 mmHg with concurrent anaphylaxis occurring after vaccination, and three of the nine patients with anaphylaxis developed HA. Although two of the three had a history of hypertension, the present patient never had hypertension and had no psychiatric pathology. Because the auscultation in the chest was not wheezing, but rather stridor accompanied by angioedema, it did not show exacerbation of BA. On the patient’s report, the basal BP before vaccination showed that the patient was normotensive. The mental condition was also stable after vaccination. Moreover, the radical and multiple clinical symptoms of stridor, angioedema, nausea, conjunctival hyperemia, and facial swelling followed by increased blood pressure which were caused in a short time indicated a type I hypersensitivity reaction such as anaphylaxis. Moderna COVID-19 vaccination causes HTN [7], but in the present case we experienced HTN that was not directly caused by vaccination. Anaphylaxis and acute increased blood pressure developed at the same time, and BP was corrected after treatment for anaphylaxis. Moreover, other diseases which cause increased blood pressure had been excluded as differential diagnoses; therefore, HA was diagnosed. Although awareness is low, it is necessary to recognize the possibility of HA occurrence to allow for early diagnosis and treatment.

The Moderna COVID-19 vaccine ingredients are messenger ribonucleic acid as the active ingredient, acetic acid, lipids, polyethylene glycol (PEG), sodium acetate, sucrose, tromethamine, and tromethamine hydrochloride. Of these ingredients, PEG is the excipient mostly incriminated for allergic reactions [8,9]. The present case was a female; similarly, previous reports of Moderna vaccination anaphylaxis were mostly in females. It has been pointed out that transcutaneous sensitization to PEG contained in cosmetics may account for this finding [10]. Regarding age, the median age of patients with anaphylaxis was 40 years, and 90% of the reported cases occurred in women [11]. Moreover, anaphylaxis in older people was often more severe than in younger adults [12]. The duration between vaccination and the administration of the first dose of AD in the present case was more than 30 minutes. This was because the multi-organ symptoms of anaphylaxis did not develop immediately and were preceded by HTN. The acute onset of increased blood pressure in the present case may have been a prodrome of anaphylaxis. BP increased until anaphylaxis treatment was administered and transiently increased after AD injection; however, BP improved approximately 30 minutes thereafter. Although there are a few case reports about HA [3,13], it may occur in anaphylaxis induced by an allergen and not only in Moderna COVID-19 vaccination. It was reported that the rate of HA among anaphylaxis patients was 12.9%. Increased blood pressure may occur alongside anaphylactic reactions due to compensatory vasopressor responses [3]. Thus, anaphylaxis may not only present with hypotension but may also be present with increased blood pressure in some cases. During anaphylaxis, the immediate release of a series of mediators, including histamines, leukotrienes, prostaglandins, thromboxane, and bradykinins from basophils and mast cells, cause increased mucous membrane secretions, increased capillary permeability, and leakage, and markedly reduced smooth muscle tone in blood vessels [14].

The mechanism of HA occurrence is not yet known; however, internal compensatory vasopressor responses such as secretion of catecholamines, activation of the angiotensin system, and production of the potent vasoconstrictor peptide endothelin-1 may induce unsteady reactions and increase peripheral resistance to high levels during anaphylactic episodes in several patients [14]. Serotonin also has an important role in the mechanism of anaphylaxis and may induce systemic hypertension through its receptors [15,16]. Blood platelet serotonin 2A receptor (HTR2A) located on vascular smooth muscle, endothelial cells, and cardiomyocytes regulate blood pressure and heart rate, HTR2A is also involved in hypertension due to its vasoconstrictive effect on peripheral vasculature [17]. It is important to note that both anaphylaxis and increased blood pressure can be treated by AD injection. Epinephrine, which down-regulates histamine released from mast cells and other mediators of inflammation, is an option for the treatment of anaphylaxis and should be injected promptly in cases of anaphylactic reactions [18]. The presence of cardiovascular disease does not exclude the use of AD in anaphylaxis because no other medications match the life-saving effects of this drug in this medical emergency [19]. More cases of HA need to be studied and verified. To the best of our knowledge, this is the first report which demonstrates the possibility of HA after Moderna COVID-19 vaccination. HA occurs rarely; therefore, most physicians are unaware of it or do not actively watch out for it. We would like to emphasize that the presence of acute onset of increased blood pressure does not rule out anaphylaxis; there is a need to promptly administer intramuscular AD injection even in HA without hesitation to save lives.

## Conclusions

This is a rare case report of Moderna COVID-19 vaccination induced HA. Although the detailed mechanism is still unclear, acute onset of increased blood pressure after vaccination may be a premonitory symptom of anaphylaxis. An understanding that HA following COVID-19 vaccination could occur in some instances is important for making the diagnosis and treating patients early to save lives.
